# Department of Canine, Feline, and Avian Medicine and Surgery

**Published:** 1901-07

**Authors:** Cecil French

**Affiliations:** Washington, D. C.


### DEPARTMENT OF CANINE, FELINE, AND AVIAN MEDICINE AND SURGERY.

By Cecil French, D.V.S.,
WASHINGTON, D. C.

---

A Case of Bilateral Cryptorchism. Cryptorchism is not uncommon in the dog. Most authors describe the unilateral form as being most common. In my experience, the malformation has usually involved both glands, and the latter have generally found lodgement within the abdominal cavity. The term " ectopia testis " has been employed to describe the condition of lodgement beneath the skin next to the glans penis.

It is well known that a retained testicle may grow to some extent at puberty, yet neither then nor at any subsequent period does it reach its full size and acquire its spermatozoa-producing powers, the tubules remaining atrophied. While incapable of producing spermatozoa, the retained organ is capable of exerting that influence which the normal testis exerts upon the development of the penis and growth of the body. When one organ has fully descended and developed, the animal is capable of procreation, but, on the other hand, when both have failed to reach the scrotum it remains sterile.

Griffiths (*Journal of Anatomy and Physiology*, London, 1892–93, xxviii., p. 209) experimented on several dogs, both young and full-grown, by replacing the testicle and cord in the abdominal cavity, with the following results :

1. When replaced in young animals it undergoes little change, growing somewhat, but not so much as the undisturbed organ until the approach of puberty.

2. The testicle so replaced after the onset of puberty continues to grow to some extent, though but little.

3. The testicle of a full-grown animal, when replaced, soon dwindles to two-thirds or one-third its natural size, and after a short time presents precisely the same structure as that which is found in the replaced testicle of the young animal. There is no trace of spermatogenesis in the degenerated epithelial cells, and no spermatozoa in the interior of the atrophied tubules.

The instance I am about to communicate was one of ectopia in a two-year-old dog—that is to say, both testes had traversed the inguinal canal and reached the position normally occupied by the scrotum, but the latter was absolutely wanting. The glands could be distinctly made out by palpating the skin, and on section proved to be possessed of all the adjunctive parts (processus vaginalis, etc.), though themselves were somewhat atrophied. The two most interesting points about the case were: the entire lack of sexual proclivity and the rudimentary condition of the penis.

I tried the animal with three different rutting bitches, but it had no idea whatever of sexual intercourse. The penis was about one-third its natural size, pale and flaccid.

This case demonstrates that not only is a retained testicle incapable of reaching maturity, but one that has traversed the inguinal canal and almost reached its goal may remain in a similar rudimentary condition.

---

THE intravenous or subcutaneous injection of normal salt solution is the latest therapeutic agent recommended in human practice for the treatment of tetanus.

ALCOHOL is now listed among the antidotes to carbolic acid poisoning.

THE last report of the United States Commissioner of Education states that there are in this country thirteen veterinary colleges with an attendance of 316 students.

FORMATION OF CINCHONA ALKALOIDS. These are, according to the investigations of J. P. Lotsey, formed in the leaves of the tree. From this location they are carried in solution to the stems, and reach a final resting-place in the bark. His observations convince him that they are formed by direct synthesis as a result of the action of cinchonic acid or ammonia, or a compound of ammonia and subsequent condensation.

